# Cytotoxic Lactalbumin-Oleic Acid Complexes in the Human Milk Diet of Preterm Infants

**DOI:** 10.3390/nu13124336

**Published:** 2021-11-30

**Authors:** Katherine E. Chetta, Joseph L. Alcorn, John E. Baatz, Carol L. Wagner

**Affiliations:** 1 Department of Pediatrics, Division of Neonatal-Perinatal Medicine, Medical University of South Carolina, Shawn Jenkins Children’s Hospital, 10 McClennan Banks Drive, MSC 915, Charleston, SC 29425, USA; baatzje@musc.edu (J.E.B.); wagnercl@musc.edu (C.L.W.); 2 Department of Pediatrics, Division of Neonatology and Pediatric Research Center, The University of Texas Health & Science Center at Houston, 6631 Fannin Street MSB 3.252, Houston, TX 77030, USA; Joseph.L.Alcorn@uth.tmc.edu

**Keywords:** human milk, HAMLET, bioactive proteins, pasteurization, inflammation, NF-κB pathway, cytotoxicity, necrotizing enterocolitis

## Abstract

Frozen storage is necessary to preserve expressed human milk for critically ill and very preterm infants. Milk pasteurization is essential for donor milk given to this special population. Due to these storage and processing conditions, subtle changes occur in milk nutrients. These changes may have clinical implications. Potentially, bioactive complexes of unknown significance could be found in human milk given to preterm infants. One such complex, a cytotoxic α-lactalbumin-oleic acid complex named “HAMLET,” (Human Alpha-Lactalbumin Made Lethal to Tumor cells) is a folding variant of alpha-lactalbumin that is bound to oleic acid. This complex, isolated from human milk casein, has specific toxicity to both carcinogenic cell lines and immature non-transformed cells. Both HAMLET and free oleic acid trigger similar apoptotic mechanisms in tissue and stimulate inflammation via the NF-κB and MAPK p38 signaling pathways. This protein-lipid complex could potentially trigger various inflammatory pathways with unknown consequences, especially in immature intestinal tissues. The very preterm population is dependent on human milk as a medicinal and broadly bioactive nutriment. Therefore, HAMLET’s possible presence and bioactive role in milk should be addressed in neonatal research. Through a pediatric lens, HAMLET’s discovery, formation and bioactive benefits will be reviewed.

## 1. Introduction

Human milk used in neonatal intensive care commonly undergoes freezing and thawing, and must be pasteurized if donated through a certified milk bank [1]. Freshly expressed milk has minimal unesterified or “free” fatty acids (FFA) which make up less than <0.5% of total fat content [2,3]. Yet freezing human milk can inherently alter the milk fat membrane globule, damaging the barrier that protects triglycerides from the hydrolytic processes of bile salt-stimulated lipase and lipo-protein lipase before ingestion [4]. Active lipases increase total FFAs [5,6]. Milk lipases continue to hydrolyze triglycerides even in −20 °C storage, further increasing levels of FFA over storage time [7,8]. Pasteurization can also increase lipolytic activity [8]. Additionally, preterm milk has increased proteolytic enzymes, such as plasmin, that may pre-digest proteins [9,10]. Heat treatment by pasteurization, and more so retort processing (sterilization), denatures proteins, which in turn influences protein bioavailability and bioactivity [11,12,13,14,15]. Conformational changes of the most abundant whey protein, alpha-lactalbumin (ALA), can increase the hydrophobicity of the molecule, making it more likely to bind to FFA and casein micelles [14,16]. One such bioactive complex has already been elucidated.

The milk-derived protein-lipid complex named HAMLET (Human Alpha-Lactalbumin Made Lethal to Tumor cells) was found in human milk in 1995, and is a complex of unfolded ALA and oleic acid. The protein was discovered serendipitously while studying cell adhesion factors using human milk [17]. Several methods have now been verified to create HAMLET in situ, including exchange chromatography, heating components in solution and even simple mixing. Homologous protein-oleic acid complexes from other species have formed cytotoxic products from cows (BAMLET), camels (CAMLET) or goat (GAMLET) [18,19,20]. The reports of apoptosis in transformed and non-transformed immature cell lines in cell culture prompted the questions: How does HAMLET affect the immature preterm intestine? What is its biological and/or evolutionary role? The presence of HAMLET in fresh preterm milk or donor milk has not been described. HAMLET’s effect on immature intestinal cells is currently unreported. Here we will discuss HAMLET’s potential role in the neonatal diet.

## 2. Discovery of a Cytotoxic Milk Complex

The original crude HAMLET complex was isolated by fractionating human milk casein using an anion exchange column [17]. FFA were then released from the column with a hydrophobic buffer, and identified as stearic, myristic, and oleic acid, indicating that multiple lipids could bind to ALA [21]. Only the oleic acid, but not other fatty acids, bound to ALA to produce cytotoxic effects [16]. When adding equivalent concentrations of oleic acid and HAMLET to experimental conditions, oleic acid alone does not have in situ cytotoxicity [22].

A calcium cofactor stabilizes internal disulfide bonds of native ALA [23,24]. When unfolded by Ca^2+^ chelation, ALA loses its calcium cofactor and forms an apoprotein. This more globular partially unfolded form, also known as a “molten state,” can readily bind with fatty acids, unlike the Ca^2+^-bound state [16,21]. The alpha-lactalbumin alpha-helix residues avidly participate in the complex interaction with oleic acid, with possible specificity for oleic acid over other fatty acids [16]. HAMLET was found to be structurally diverse depending on the method of formation. Due to its globular form and strong tendency to aggregate, HAMLET has so far defied characterization by X-ray crystallography and NMR but has recently been characterized by tandem mass spectrometry and small angle X-ray scattering [25,26]. It exists primarily as a monomeric protein (with 5% dimers or higher order multiples); however, higher-heat treatments can form a greater concentration of the multimeric form. HAMLET naturally forms aggregate structures of protein with 1–12 proteins and 50–100 molecules of fatty acid, and even monomers can carry more than 20 molecules of fatty acid depending on the method of preparation [27]. HAMLET-like complexes can also be formed from digested ALA fragments, suggesting that the alpha-helix domain is the key unit which associates with oleic acid [26,28]. Unfolded ALA can also bind with mono-unsaturated fatty acids, but only oleic acid (C18:1ϖ9 or C18:1ϖ11) is cytotoxic [16]. The exact bonding mechanism between apo-state ALA and free oleic acid is still unknown, and it is likely that hydrophobic interactions and van der Waals forces are involved.

### 2.1. Bioactivity of HAMLET

Recently, the specificity of HAMLET to cancerous cells alone has been called into question by a careful comparative study of HAMLET lethal doses against multiple cell lines [22]. HAMLET cytotoxicity is variable depending on cell type and experimental conditions in which HAMLET is formed. The cytotoxic-specificity of human protein is considered up for debate as similar compounds have been made from milk of various animal species, though HAMLET from human milk may be most cytotoxic. A recent study compared HAMLET’s cytotoxicity with that of free oleic acid alone by directly measuring free fatty acid content. The authors reported that 10 times the molar equivalent of pure oleic acid was needed to generate the same degree of apoptosis as the complex. They concluded that the ALA apo-protein helped the inherently cytotoxic oleic acid dissolve in solution at normal pH [29]. “Crude HAMLET” or multimeric alpha-lactalbumin (MAL) isolated from human milk required concentrations of 10 mg/mL for cytotoxicity [17]. Pure synthesized BAMLET (HAMLET from bovine milk) has a lethal dose of 50–150 µg/mL, but is highly dependent on cell type and culture conditions [22]. It is currently unknown how much HAMLET occurs naturally in human milk.

Nonetheless, a complex interplay between free oleic acid levels and protein cannot be easily excluded. An elegant study by Hakansson et al. showed that biotinylated HAMLET differentially enters the cell and targets the nucleus of A549 cells as compared to native ALA [30]. The cytotoxic properties of HAMLET are theorized to be attributed to the multiple oleic molecules in conformation, as if increasing the potency and directing the target of free fatty acids. Yet, the specific components of the ALA protein are also important. It was recently shown that the alpha-helix protein fragments of ALA, but not the beta-sheet leads to cell cytotoxicity in the presence of oleic acid [26].

Additionally, HAMLET is a pan-kinase inhibitor. Using a proteomics-based microarray of over 8000 human proteins coupled with a HotSpot high-throughput radiometric assay, HAMLET was shown to bind to various nucleoside binding proteins and inhibit 69% of kinases. HAMLET showed high associations with ATP-binding (28.55%) and GTP-binding domains (6.45%) [31]. Therefore, HAMLET’s effect on living tissue may reach far beyond its cytotoxic properties.

If HAMLET and/or dissolved free fatty acid could cause milk cytotoxicity, there may be important implications for extremely preterm infants, especially when considering the rise in total FFA over storage time. HAMLET has been postulated to form spontaneously in human milk; therefore, various hypothetical harms and benefits may be associated.

### 2.2. Prevalance and Digestion of HAMLET

In general, the type of human milk used to isolate HAMLET is not well characterized. The first published HAMLET/MAL study simply reported “breastmilk casein” [17], no further information was given about the source, storage duration, or the general treatment of the milk before use. Describing human milk that contains HAMLET could give insight into whether this protein is found naturally in fresh expressed milk or is formed after freezing or pasteurization. It is unknown whether HAMLET is higher in term or preterm milk.

It has been suggested that normal digestion could catalyze the formation of HAMLET in vivo. There are a few problems with this theory: (1) the pH ranges needed to unfold ALA in experimental conditions to synthesize HAMLET are within alkaline ranges of 8-10. No authors have demonstrated HAMLET synthesis in low pH conditions; (2) While gastric acidity of pH values < 4 may aggregate ALA [32], free fatty acid may not be available because lipase activation conversely decreases in acidic conditions. Therefore, even if ALA was unfolded by a low pH, lipase may be inactive in this state to free sufficient fatty acid. In a study of 15 preterm infants, gastric acidity was observed over feeding time using multipoint intragastric pH monitoring. In this study, the majority of the feed buffered the acidity of the stomach and maintained a pH of 5–7 [33].

In summary, it is unknown whether HAMLET could survive digestion to affect the intestinal environment, and/or if it forms in digestion spontaneously. It is possible that the structure of HAMLET could help it pass through the stomach intact. A recent small-angle X-ray scattering study revealed that HAMLET’s structure resembles a protein-coated sphere with a fatty acid core [34]. A fascinating article reported that alpha-lactalbumin, when exposed to certain proteases, could spontaneously form nanotube structures. Currently, these nanotubes are being used for pharmaceutical delivery, for example to trap bioactive ingredients for slow release in to the intestine [35]. Could HAMLET behave similarly to these nanotube structures which could deliver fatty acids or other hydrophobic substances deep into the gastrointestinal tract? Protein labeling, imaging (like transmission electron microscopy) coupled with animal models could help answer these questions.

## 3. Conditions for HAMLET Formation in Human Milk

### 3.1. Freezing and Pasteurization Releases Free Oleic Acid

There are several processes that may lead to formation of HAMLET in human milk, especially in milk for hospitalized or preterm infants. One of the likely contributors is the naturally high oleic acid content of human milk. The predominant form of milk fat (>99%) is in the form of triglycerides, with oleic acid being the most common fatty acid observed in large population-based studies performed around the world [36,37,38]. Naturally occurring FFA is minimal in freshly expressed human milk, even though bile-salt stimulated lipase and lipoprotein lipase are also present. The milk fat globule membrane provides a natural barrier between triglycerides and lipases, but freezing milk fractures the membrane, disrupting the globule and freeing triglycerides. Lipases begin to digest triglycerides and release FFA before consumption by the infant [4,39]. This is important because HAMLET complex formation is highly associated with the amount of FFA present [22]. Pasteurization can deactivate lipase, theoretically preventing the time-dependent release of FFA, but FFA may be present before donation. In some banks, frozen milk can be received up to eight months after expression (Human Milk Banking Association of North America correspondence). Therefore, multiple theoretical avenues during human milk handling and storage could generate significant FFA for HAMLET formation.

### 3.2. Pasteurization Changes Alpha-Lactalbumin Protein

Alpha-lactalbumin is a 14kD protein, classically denatured by heat [11,13]. Before donation, milk undergoes Holder pasteurization (62.5 °C, 30 min) to neutralize harmful pathogens. Pasteurization increases denatured whey proteins in solution. Heat treatment for 10 min at 60 °C begins to aggregate ALA [40]. Conditions up to 70°C for a few seconds can denature 36% of whey protein [14,41]. Partial denaturation, through the unfolding of ALA, is the first step in the creation of HAMLET. The direct effects of retort processing and sterilization have not been studied for human milk proteins, but based on scientific evidence generated from the dairy industry, retort and ultra-high temperature processing could significantly change whey proteins leading to precipitation onto casein micelles, aggregation, and the formation of new disulfide-bound complexes [14].

### 3.3. Calcium, Phosphorus and Alpha-Lactalbumin

Casein solubilizes large quantities of calcium and phosphorus in milk. Soluble calcium levels fall after pasteurization, possibly from precipitation of calcium phosphate salts after casein micelles are disrupted [42]. It is unknown how calcium and phosphorus may precipitate in frozen storage over time. Because calcium stabilizes the native state of ALA, decreased calcium levels may catalyze the unfolding of alpha-lactalbumin [43].

## 4. Clinical Implications for Critically Ill and/or Preterm Infants

It has been suggested that because “HAMLET consists of molecules from human milk, which are ingested daily by premature and newborn infants, toxic side effects are unlikely” [44]. However, critically ill newborns and extremely preterm newborns have immature and fragile intestinal environments and commonly suffer from severe digestive diseases including necrotizing enterocolitis (NEC), spontaneous intestinal perforation and recurrent feeding intolerance. Because this population is usually unable to feed directly at the breast, milk is pumped and frozen. In general, mother’s milk is the preferred mode of feeding infants and is significantly more protective against intestinal disease like NEC than formula feeding [45]. Very preterm babies that do not have their mother’s milk will receive pasteurized donor human milk in most high-level units (>Level 2) in the US [46]. The incidence of NEC has decreased over the last decade as strategies for prevention have evolved [47], but it remains the most common gastrointestinal emergency in the NICU and continues to warrant more research for both treatment and prevention. The growing utilization, storage and engineering of human milk generates a critical and timely opportunity for investigation.

### 4.1. Inflammation and Necrotizing Enterocolitis

Many preterm diseases are characterized by high inflammatory states in one or more organ systems. Unfortunately, the incidence of severe NEC in the US remains at 2–4%, affecting up to 10% of infants with a birth weight < 1000 g [47,48]. The mortality rate of severe NEC in the very low birth weight population approaches 50%. While the cause of NEC is multifactorial, its pathophysiology is linked to overactive inflammatory signaling in the intestine [49,50]. Contributing factors include pathogenic microorganisms, a dysregulated microbiome, dysfunctional intestinal barriers, formula feeding, and episodes of hypoxia/reperfusion injury. The strongest risk factor for NEC is prematurity, which emphasizes that the disease is highly specific to a critical developmental timeframe [51]. Preventing NEC is not possible with a single intervention; rather, multiple strategies can decrease inflammation and limit its incidence and severity. One of the most effective preventative strategies is the use of human milk [52]. Donor milk, while still protective, is less so than maternal milk [53]. Could HAMLET, which could possibly be generated by processing and freezing, contribute to the relative risk and severity of NEC in pasteurized or frozen milk samples?

#### 4.1.1. Relevant Animal Studies

Early animal studies reported that the degree of injury in a porcine model of NEC worsened in the presence of lipids in the enteral diet. Defatted formulas led to less severe NEC in piglets with reperfusion injury than with full fat formulas [54]. Bovine casein protein also has been repeatedly linked to NEC [55,56]. Di Lorenzo reported that acidified casein solution increased intestinal inflammation in piglet intestines after three hours [57]. Certain milk macronutrients, when disrupted from the natural enveloped system of a micelle or globule, may cause inflammation through various factors such as FFA. Whether these disrupted components have biologic relevance as a synergistic whole deserves further investigation.

#### 4.1.2. Inflammation and HAMLET: In Vitro Evidence

The preterm intestine is characterized by upregulated inflammatory signaling, including excessive responses to stimulation of the TLR-4 and IL/1β with NF-κB pathways. The pathogenesis of NEC on a tissue level is characterized by increased transcription of IL-8, TLR2, TLR4, MyD88, TRAF-6, and NFκB2, when comparing intestinal resection from NEC and healthy controls [58,59]. In studies with healthy lymphocytes, HAMLET but not native ALA was shown to significantly upregulate NF-κB expression. HAMLET increased IL-6, IL-8 and TNFα, even localizing in proximity to TLR-4 receptors [60]. The cytokine profile of infants with NEC is similar, demonstrating increased enterocyte apoptosis secondary to upregulation of TLR-4, and subsequent increases in IL-6 and IL-8. Vansarla et al. reported that HAMLET primarily signals through NF-κB pathways. The NF-κB pathway plays a prominent role in NEC and when inhibited, can also decrease phenotypic severity [59]. The overlap between signaling pathways of HAMLET and the upregulated pathways identified in NEC are concerning; however, other factors in human milk may mitigate the inflammatory effect. A recent evaluation of probiotic effects on colonic cells showed inhibited NF-κB pathways as a possible mechanism of protection against NEC [61]. No studies have investigated HAMLET’s effect on intestinal crypt cells, fetal gut epithelial cells, or other relevant models of the preterm gut. If the quantity of HAMLET in milk is increased by milk storage, the balance of inflammatory and anti-inflammatory factors could be dysregulated.

#### 4.1.3. Oleic Acid Content Is Modifiable

Interestingly, one of the few modifiable factors of human milk is the fatty acid profile [62]. A maternal diet high in animal fats can contribute to increased oleic acid in human milk, as compared to a fat-free or vegetable-fat diet. When comparing the milk produced by Chinese women from a marine area (with high seafood intake) against other areas (urban/rural/pastoral) the oleic acid content of human milk was significantly lower [63,64]. Evidence is growing in animal models that a western maternal diet can increase neonatal inflammation [65]. More evidence is needed to determine specific effects of maternal diets on neonatal inflammation and outcomes. Inflammation is increasingly linked to poor growth outcomes as well as poor neonatal neurological outcomes [66]. Whether spontaneous HAMLET formation in milk may be affected by maternal diet is unknown.

### 4.2. HAMLET, Donor Milk and Infant Somatic Growth

Preterm infants are at high risk for extrauterine growth failure [45,67]. Randomized trials show that donor milk feeds are associated with slower growth than formula, even when traditionally fortified with bovine products. Despite this, donated human milk is currently the optimal nutrient source for the preterm infant if mother’s milk is unavailable because it reduces the risk of NEC [68]. A lack of appropriate fortification does not explain the poor growth of infants on donor milk. It is possible that poor growth from donor milk may be mediated by deactivated gastrointestinal enzymes, e.g., lipases, or by the loss of fat which adheres to plastic containers and tubing [69]. However, even the addition of recombinant lipases to pasteurized human milk did not significantly improve growth outcomes [70]. Many factors likely contributing to poor growth of infants using donor milk are related to losses of constituents after pasteurization. Milk has reduced antioxidant capacity after approximately six months in frozen storage [71]. However, the bioactivity of new complexes has not been fully considered. Could HAMLET’s ability to broadly inhibit ATPase and GTPase impact cellular growth? It is possible that HAMLET could increase inflammation in rapidly growing stem cells, and its biological activity in an environment characterized by rapid growth is unknown. If HAMLET has a biological effect on growth and is formed in storage, current milk handling guidelines in the NICU could be adapted to optimize milk storage recommendations.

### 4.3. HAMLET and Potential Benefits for Premature Infants

A balanced microbiome is key to intestinal health [72]. A recent Cochrane review suggests probiotics likely decrease rates of NEC [73]. Additionally, prolonged antibiotics and the absence of early human milk feeding can disrupt the commensal environment [74,75]. HAMLET has been shown to kill streptococcus and can even enhance the activity of antibiotics [76,77,78]. Activity against Streptococcus pneumoniae, Streptococcus pyogenes and Streptococcus agalactiae (GBS) has been reported [79]. Interestingly, in an attempt to remove adulterated lipopolysaccharides from HAMLET, one laboratory reported that the fatty acid content of HAMLET was reduced by 60%, which also led to reduced bioactivity against streptococcus by 3.75x [60]. It is unknown whether HAMLET may be present in sufficient quantities to significantly affect the gut microbiome or protect the neonate from pathogenic bacteria. Even with HAMLET-like complexes potentially present in donor milk, donor milk is linked to lower NEC rates as compared with formula [68]. If HAMLET is found in donor milk, its presence may affect the microbiome.

## 5. Conclusions and Call for Neonatal Research

HAMLET is a versatile lactalbumin-based structure complexed with oleic acid leading to potent cytotoxicity specifically against immature and cancerous cells—through a mechanism which is not fully understood. Further studies are needed to investigate the clinically significant spontaneous formation of cytotoxic ALA complexes in human milk and potential (possibly unwanted) effects related to the inflammatory pathways in preterm infants. Potential areas of research include detecting HAMLET in various types of human milk and studying the processes acting as a catalyst to formation (e.g., freezing, heating, time in storage). Physiological effects could be explored through analysis of the stool or milk microbiome, and studying the effect of this lipid-protein complex on novel neonatal intestinal models. Eventually, exploring the effect of HAMLET on gut permeability or inflammation would be important.

Collaborating pediatric researchers could rapidly elucidate the biological or immunological role of HAMLET in the diets of preterm infants. This complex has intriguing features but may have deleterious effects on the immature intestine. Investigating HAMLET’s biological role and understanding the various factors involved in its formation may lead to improved outcomes of this population.
